# Unilateral Renal Ischemia-Reperfusion as a Robust Model for Acute to Chronic Kidney Injury in Mice

**DOI:** 10.1371/journal.pone.0152153

**Published:** 2016-03-23

**Authors:** Nathalie Le Clef, Anja Verhulst, Patrick C. D’Haese, Benjamin A. Vervaet

**Affiliations:** Departement Biomedical Sciences, Laboratory of Pathophysiology, University of Antwerp, Wilrijk (Antwerp), Belgium; Institut National de la Santé et de la Recherche Médicale, FRANCE

## Abstract

Acute kidney injury (AKI) is an underestimated, yet important risk factor for development of chronic kidney disease (CKD). Even after initial total recovery of renal function, some patients develop progressive and persistent deterioration of renal function and these patients are more likely to progress to end-stage renal disease (ESRD). Animal models are indispensable for unravelling the mechanisms underlying this progression towards CKD and ESRD and for the development of new therapeutic strategies in its prevention or treatment. Ischemia (i.e. hypoperfusion after surgery, bleeding, dehydration, shock, or sepsis) is a major aetiology in human AKI, yet unilateral ischemia-reperfusion is a rarely used animal model for research on CKD and fibrosis. Here, we demonstrate in C57Bl/6J mice, by both histology and gene expression, that unilateral ischemia-reperfusion without contralateral nephrectomy is a very robust model to study the progression from acute renal injury to long-term tubulo-interstitial fibrosis, i.e. the histopathological hallmark of CKD. Furthermore, we report that the extent of renal fibrosis, in terms of *Col I*, *TGFβ*, *CCN2* and *CCN3* expression and collagen I immunostaining, increases with increasing body temperature during ischemia and ischemia-time. Thus, varying these two main determinants of ischemic injury allows tuning the extent of the long-term fibrotic outcome in this model. Finally, in order to cover the whole practical finesse of ischemia-reperfusion and allow model and data transfer, we provide a referenced overview on crucial technical issues (incl. anaesthesia, analgesia, and pre- and post-operative care) with the specific aim of putting starters in the right direction of implementing ischemia in their research and stimulate them, as well as the community, to have a critical view on ischemic literature data.

## Introduction

Despite decades of scientific research, chronic kidney disease (CKD) still has an increasing incidence and prevalence [1]. In addition, it is becoming increasingly clear that acute kidney injury (AKI) is an underestimated, yet important risk factor for the development of CKD [2]. Long-term follow-up studies (4 months to 6 years) report that between 35 and 71% of patients surviving an episode of AKI had incomplete recovery of renal function as assessed by creatinine clearance or serum creatinine measurements [3]. Even after initial total recovery of renal function, some patients develop progressive and persistent deterioration of renal function [4]. Moreover, these patients are more likely to progress to end-stage renal disease (ESRD) compared to patients without a history of either AKI or CKD [2]. Getting insight in the mechanisms underlying the progression from acute to chronic renal injury is a major focus of recent research in the field [5]. Since the pathogenesis of acute to chronic renal injury involves a complex multi-cellular interplay within the heterogeneous renal tissue, animal models play a crucial role in unravelling these complexities towards development of new and efficient therapeutic modalities [6].

Rodent (mouse and rat) disease models are favourable, for several reasons: 1) widely available, 2) relative low cost as compared to higher order mammals, 3) the possibility of inducing genetic modifications, which allows both testing the role of specific proteins as well as tracking the fate of cells in disease [7,8]. Renal ischemia reperfusion injury (IRI) is one of the most used animal models for both fundamental and therapeutic intervention studies in AKI. Yet, despite the nephropathological relevance of ischemia, only a few studies applied IRI to study long-term sequelae of an acute ischemic insult [9–13]. The potential of this model as an initiator of CKD has not been systematically investigated. The IRI model comes in different flavours, each with their own natural course of renal dysfunction and histopathology. Importantly, not all IRI variants are suited to study the progression from AKI to CKD and fibrosis. A distinction has to be made between cold and warm renal ischemia reperfusion. Cold ischemia, where ischemia is either performed at 32°C body temperature [14,15] or by cooling the kidney to 4°C [16–18], is a rarely used variant of the IRI model. Most often cold ischemia actually refers to cold storage of the kidney before transplantation, either with [19–21] or without [22–24] warm ischemia prior to the cold storage period. Warm ischemia, on the other hand, is most frequently used and can be subdivided into bilateral ischemia-reperfusion (BIR) and unilateral ischemia-reperfusion (UIR). Depending on the presence of the contralateral kidney, UIR can be further subdivided into UIR without contralateral nephrectomy and UIR with contralateral nephrectomy. An additional variation on the latter model consists in the timing at which the uninjured contralateral kidney is removed (cfr. Skrypnyk et al. [25]).

As a model of AKI, bilateral ischemia-reperfusion injury (BIRI) affects total renal mass and induces a measurable increase in serum creatinine and blood urea nitrogen (BUN), both functional hallmarks of AKI in patients [26]. However, with respect to its application for studying chronic renal fibrosis, a strict control of the severity of the induced ischemic renal injury is critical: when renal injury is too mild, near complete recovery of the kidneys ensues without progression towards chronic renal injury and fibrosis [27,28]. On the other hand, when the induced ischemic insult is too severe, animals are very likely to die of acute renal failure within 48 hours [25]. Although long-term studies with BIRI have been performed [3,29,30], most studies indicate that kidney morphology returns to almost normal 2 weeks after the bilateral ischemic insult. A few studies reported a limited number of tubules with signs of damage and some lymphocyte infiltration in the interstitium [29,30]. Microvascular rarefication was noted to be present 4 weeks after BIRI [31] and some glomerular atrophy and hypertrophy and interstitial scarring was observed 40 weeks after BIRI [3]. Serum creatinine returned to sham-levels 16 days after BIRI and remained stable up to 40 weeks after BIRI [3,31], indicating no long-term functional decay. The pathological course of unilateral ischemia-reperfusion injury (UIRI) with immediate contralateral nephrectomy (i.e. during the same surgery) is expected to be quite similar to BIRI in the sense that in both models, the animals leave the surgical procedure with injured renal tissue only. On the other hand, in UIRI without contralateral nephrectomy, animals still have a healthy kidney left in place. Due to this functional redundancy, the risk of mortality caused by acute renal failure is highly reduced [27] and the consequences of AKI can be investigated well beyond the first days of acute injury [32]. Furthermore, UIRI without nephrectomy allows to conduct longer ischemia times [29] (up to 60 minutes in mice [9] and 190 minutes in rats [33]), thus allowing studies in a larger range of severity of kidney injury. This potential of the UIRI model without nephrectomy in inducing a range of histopathological renal injury more closely resembles the variability in nephropathology seen in patients [34]. Moreover, development of an uremic milieu, as occurs with BIRI, and which is part of the renal pathology in patients, is avoided [26].This allows the assessment of the natural course of post-ischemic renal damage without the possible protective effects of uraemia, i.e. cytoprotective [35] and anti-inflammatory effects [36]. It should be noted, however, that due to the presence of the non-injured contralateral kidney, the functional course after UIRI cannot be assessed by simply taking a blood sample and collect 24-hours urine to calculate creatinine clearance [25]. One way is to remove the healthy contralateral kidney and measure glomerular filtration rate (GFR) hours after removal [11]. However, depending on the severity of the acute ischemic insult, this can be an end-point analysis. Alternatively, split renal function measurement by use of ureter catheterization has been performed in dog [37,38], pig [39] and rat [40,41]. In rats, however, this is done shortly before euthanasia (end-point analysis), and this technique is unlikely to be suitable for mice.

Generally, consequences of AKI induction through ischemia-reperfusion are studied 24–48 hours up to 2 weeks after the insult [11,28,42]. However, we hypothesize that the model of UIRI without nephrectomy is valuable to study the evolution of the histopathology of acute ischemic kidney injury progressing to CKD with long-term development of fibrosis. In the present study we therefore investigated the long-term renohistopathological outcome of UIRI (without nephrectomy) with emphasis on evaluating the development of fibrosis whilst varying the two most important determinants of ischemic injury, i.e. core body temperature during and duration of ischemia. Since we studied AKI-induced CKD, we also included analysis of long-term expression of tubular injury markers and inflammatory cytokines. In addition, despite the intuitive simplicity of the IRI model, i.e. obstruct renal blood flow for a given period of time, many technical factors influence the renal pathological outcome, making this model in its execution more complicated and less reproducible than generally anticipated. In order to cover the whole practical finesse of ischemia-reperfusion, we complemented this manuscript with a referenced overview on crucial technical issues with the specific aim of putting starters in the right direction of implementing IRI in their research and stimulate them, as well as the nephrology community, to have a critical view on the findings of others.

## Materials and Methods

### Technical survey and considerations on the ischemia-reperfusion procedure

With the aim of providing full methodological insight, we performed a literature survey on several important practical aspects of the renal ischemia-reperfusion model, i.e. the use of anaesthesia and analgesia and the influence of ischemia time and body temperature. Since the latter two are considered to be the main determinants for fine-tuning the ischemic model, we further experimentally evaluated their impact specifically in the setting of unilateral ischemia-reperfusion.

### Experimental evaluation of unilateral ischemia-reperfusion in mice

All surgical procedures were conducted according to the National Institute of Health Guide for the Care and Use of Laboratory Animals and approved by the University of Antwerp Ethics Committee (approval number 2011–51). Sample size (n = 6) was determined by power analysis with respect to the 3R principle of animal ethics. Some UIRI conditions were repeated to verify reproducibility, i.e. UIRI at 35°C for 30 minutes and at 36°C for 21 and 18 minutes. On average, we encountered 6% mortality, mainly due to post-anaesthetic complications. In addition, on average 8% of the animals were excluded from analysis, because its values were marked as outliers for different parameters upon statistical analysis. Prior to surgery, animals were randomly allocated into the different groups. Animals had free access to standard chow and tap water.

#### Study set-up

Male C57Bl/6 mice (10–12 weeks of age; Charles River, Saint-Germain-Nuelles, France) subjected to an acute ischemic kidney injury by unilateral ischemia-reperfusion (UIR) without contralateral nephrectomy consistently develop post injury renal fibrosis. As already mentioned above, the extent of acute renal injury is dependent on both body temperature and ischemia time [43]. To examine (1) the effect of body temperature during ischemia on fibrotic outcome after UIRI, the left kidney was clamped for 30 minutes at 37°C (n = 5), 36°C (n = 4), 35°C (n = 10) or 34°C (n = 5) and animals were euthanized 12 weeks after UIRI; (2) the effect of ischemia time on fibrotic outcome after UIRI, the left kidney was clamped for 30, 21 or 18 minutes at 36°C and animals were euthanized 6 weeks (resp. n = 5, n = 12, n = 6) and 12 weeks (resp. n = 4, n = 5, n = 10) after UIRI.

#### Surgical procedure

**Optimized pre-operative preparation of the animal for the induction of IRI:** The mouse is anaesthetized with a mixture of ketamine (Ketalar, Pfizer, Elsene, Belgium; 80 mg/kg) and xylazine (Rompun, Bayer, Wuppertal, Germany; 16 mg/kg), diluted in sterile saline to a final volume of 2.4 ml/100 g body weight. The mixture is administered intraperitoneally whereupon the animal is immediately transferred to an incubator set at a fixed temperature until loss of righting reflex has occurred. It is of paramount importance that body temperature is monitored throughout the procedure since body temperature may decrease by several degrees following administration of ketamine and xylazine [44] and hypothermia is one of the most common causes of anaesthetic death. Following induction of anaesthesia, which usually takes 3–5 minutes, the left side of the abdomen is depilated and disinfected with 70% ethanol. Depilation of the abdomen is done with Veet cream (Reckitt Benckiser, Brussel, Belgium), followed by sufficient cleaning with moist sterile tissues to remove any cream remnants. Preferably, depilation is conducted one day in advance, for several reasons: 1) it improves working efficacy on the day of surgery, 2) it allows a more efficient time use during which the animal is sedated (45–60 minutes) 3) it avoids the need to administer additional anaesthesia, 4) it induces less skin irritation and, 5) depilation of the skin just before surgery substantially contributes to the decrease of body temperature that occurs after anaesthesia even when placed on a heating pad. Overall, depilation of the skin 24 hours in advance improves the reproducibility of the surgery. Next, eye ointment (Duratears, Alcon-Couvreur, Puurs, Belgium) is applied to make sure the cornea is protected from drying and trauma, and buprenorphine (Temgesic, Reckitt Benckiser, Brussel, Belgium; 0.05 mg/kg), diluted in sterile saline, is provided via intraperitoneal injection. The animal is placed with its back on a heating pad (Physitemp, Clifton, New Jersey) in a position with its head and neck extended to ensure that its airway remains unobstructed [45]. The body temperature is monitored trough a rectal probe, with a feedback system to the heating pad (Physitemp, Clifton, New Jersey).

Before initiating surgery, anaesthetic depth is determined by touching the medial corner of the eye, which should not result in a response and by testing the withdrawal response by applying pressure with a fingernail to the back foot of the animal, who should not withdraw [46]. Respiration should be monitored to ensure that it is of adequate depth and normal frequency [47].

**Surgery:** Surgery should not be started before the core body temperature of the mouse, as measured with a rectal probe, is stabilized at the set point and the mouse is in deep anaesthesia. Stabilization of the core body temperature can take up to 15 minutes, may require a heat lamp (in addition to the heating pad) and a draft-free location to facilitate this process. The abdomen is opened with a midline incision; approximately 1–1.5 cm. Using a wound spreader the intestines are carefully pushed aside and the left kidney is exposed, however not exteriorized to avoid rigorous cooling of the kidney and ischemic preconditioning during manipulation of the kidney. The renal pedicle is carefully dissected with fine-point tweezers to remove the perihilar adipose tissue, exposing the blood vessels for renal pedicle clamping. It is important that the blood supply to the adrenal gland remains unaffected. The renal pedicle is clamped with an atraumatic vascular clip (Scanlan, Saint Paul, Minnesota; see study set-up for the indicated ischemia times and body temperature used in the experiments described in this manuscript) using holding forceps, ensuring minimal vascular damage, and as little as possible perihilar fat in the clamp. Successful ischemia is characterized by a gradual colour change of the kidney from red to dark purple within 1–2 minutes. The right kidney is left undisturbed. The abdomen is temporarily closed with a suture and the animal is transferred to an infant incubator, kept at the temperature that allows the body temperature of the animal to remain stable at the set-point temperature for the duration of ischemia (see study set-up). Temperature of the animal is monitored continuously with a rectal thermometer (Bioseb, Vitrolles, France). Variations in body temperature during ischemia should be kept less than 0.5°C, as this will increase variability to the degree of renal injury. The vascular clip is released at the desired time to start reperfusion. After verification of kidney colour to change back to red (roughly within 10 seconds), a Vicryl 4–0 suture (Ethicon, Norderstedt, Germany) is used to first close the muscle layer, followed by closing of the skin. Sham-operated animals are subjected to the exact same surgical procedure, aside from clamp placement.

**Post-operative care:** Immediately after surgery, 1 ml saline or Plasma-Lyte (Baxter, Lessen, Belgium; i.e. buffered low chloride saline solution) is given intraperitoneally to compensate for the fluid loss during surgery. Even though the superiority of Plasma-Lyte over saline is not proven, clinical reports allow assuming that high chloride solutions are associated with worse AKI as compared to low-chloride solutions [48]. The animals are kept on a warm water mat (water temperature at 37°C) until awakening after which they are placed in an open grid recovery cage under a heating lamp until full consciousness is regained. Heating lamp and cage are placed in such a way that one end of the cage maintains room temperature whilst the distance between lamp and animals is held large enough to avoid overheating. After 24 hours the animals are transferred to their housing cage.

During the first 3 days after the surgery, the animals, in addition to the standard chow, are supplemented with DietGel Recovery Purified Soft Diet for Rodents (Clear H_2_O, Portland, Maine), rich in water and sugars, to reduce the post-operative weight loss and allow faster recovery after surgery. Weekly follow up of the body weight and behaviour is performed as measures of good health.

#### Real-time PCR

Total mRNA is extracted from a pole section of the ischemic kidney (PureLink RNA Mini Kit; Life Technologies, Gent, Belgium) and converted to cDNA (High Capacity cDNA archive kit; Life Technologies, Gent, Belgium). To quantify gene expression, qPCR, based on the Taqman fluorescence method (ABI Prism 7000 sequence detection system; Life Technologies), was used. Taqman probes and primers for *GAPDH* (Mm99999915_g1), *collagen I α1* (Mm00801666_g1), *TGFβ1* (Mm01178820_m1), *CCN2* (Mm01192931_g1) *CCN3* (Mm00456855_m1), *Havcr1* (Mm00506686_m1), *Lcn2* (Mm01324470_m1), *TNFα* (Mm00443258_m1) and *IL-6* (Mm00446190_m1) were purchased from Life Technologies (Gent, Belgium). Each gene was analysed in triplicate and the expression was normalized to the reference gene *GAPDH*. Calculations were made conform the comparative CT-method.

#### Histology

Renal morphology was evaluated on ischemic kidney tissue fixated in NBF (Neutral Buffered Formalin), stained with Masson’s trichrome after post-fixation in Bouin’s fixative. Masson’s trichrome stain is the standard for visualizing fibrosis in tissue, provides a useful sense of tissue morphology, and allows evaluation of localization and severity of deposition of extracellular matrix.

For collagen I immunostaining, paraffin embedded 4 μm thick sections of ischemic kidney tissue were blocked with normal goat serum and incubated overnight with the primary antibody, polyclonal rabbit anti-mouse collagen I antibody (dilution 1/3500,Catalogue number T40777R, Lot number 20I25000, Biodesign International, Saco, Maine). After washing, sections were incubated with a biotinylated secondary antibody, goat anti-rabbit IgG antibody (dilution 1/200, PK-4001, Vector Laboratories, Burlingame, California) and subsequently incubated with avidin and biotinylated horseradish peroxidase (AB-complex, Vector Laboratories, Burlingame, California). A dark brown colour was developed with diaminobenzidine in the presence of 3% H_2_O_2_. Sections were counterstained with methyl green to visualize nuclei. Collagen I immunostaining was quantified using the Axiovision image analysis software (Carl Zeiss, Jena, Germany) and quantification was performed blinded. The area % stain represents the ratio of the summed absolute areas of staining versus the total tissue.

#### Statistics

All statistical analysis was performed with SPSS Statistics 22 (IBM, Brussel, Belgium). Data are presented as mean ± standard deviation, or as individual values. Comparisons between groups are assessed using a Kruskal-Wallis test, followed by a two-tailed Mann-Whitney U test. Values of p<0.05 are considered significant.

## Results of experiments for the evaluation of unilateral IRI without nephrectomy as initiator of CKD

### Technical survey and considerations on the ischemia-reperfusion procedure

#### Anaesthesia

General anaesthesia in laboratory animals involves loss of consciousness, loss of sensation (analgesia) and muscle relaxation [46]. An ideal anaesthetic agent is easy to administer, produces a fast and adequate immobilization, has limited side effects, and is reversible and safe for animals and operators. Unfortunately such an anaesthetic is not available, and the best drug selection is highly variable according to different experimental circumstances [47] e.g. interference with pathology.

Inhalation anaesthesia is usually preferred to injection anaesthetics. Induction and recovery of inhalation anaesthesia are rapid, safer (as it causes less cardiovascular depression) and allows accurate control over the depth of anaesthesia [45]. However, compared to injection anaesthesia, inhalation anaesthetics are counter-indicated for use during IRI as it was shown that some volatile anaesthetics confer profound protection against renal IRI by attenuating inflammation [49].

Injection anaesthetics most commonly used for laboratory mice are barbiturates, dissociative anaesthetics such as ketamine, and α2 agonists. Barbiturates such as sodium pentobarbital (Nembutal, CEVA Sante Animale) are counter-indicated for IRI, as they reduce blood flow to the kidney, secondary to lowered blood pressure, with reduced glomerular filtration rate (GFR) and urine output [46]. In addition, barbiturates have a narrow margin of safety. Dissociative anaesthetics such as ketamine (Ketalar, Pfizer) and tiletamine (Zoletil, Virbac) have a wide margin of safety, analgesic potential and prevent spinal sensitization (wind-up). Ketamine is often combined with other anaesthetic agents such as α2 agonists to improve quality of anaesthesia while reducing its side effects. Ketamine combined with xylazine (Rompun, Bayer) is the most used ketamine combination in mice, producing short surgical anaesthesia (30–45 minutes) with good immobilization and some analgesia [47]. Ketamine is metabolized in the liver, producing inactive metabolites that are excreted by the kidney [46], and as such is safe to use in mice with compromised renal function as is the case with IRI. Xylazine is also metabolized in the liver, producing inactive metabolites, however it is recommended to lower the dose in case of renal failure [46]. When used during the induction of IRI, and certainly when a healthy kidney is left in place, remnant renal function is sufficient to enable its safe use.

#### Analgesia

Analgesia should be based on the species, the type of procedure performed, the pharmacokinetics of available agents, and any known adverse effects of the specific drugs [44]. Also, it is currently believed that analgesia administered pre-operatively (pre-emptive analgesia) can provide a more efficient and readily pain control [45]. Analgesics most commonly used for laboratory mice are opioids and non-steroidal anti-inflammatory drugs (NSAIDs) [50].

Opioids are part of the most potent analgesic agents. Fentanyl (Durogesic, Janssen-Cilag), oxymorphone (Opana, Endo Pharmaceuticals), buprenorphine (Temgesic, Reckitt Benckiser) and butorphanol (Stadol, Hospira) are the most commonly used opioids in laboratory animal care. Fentanyl is the most powerful, but is also the shortest acting. In addition, it is given transdermally by skin patch, making it less favourable for being used routinely [47]. Buprenorphine seems to be the most appropriate analgesic for use in mice undergoing IRI because of its long lasting (12 hours) effect, high therapeutic index and its potential for being used in animals with compromised renal function since it is metabolized in the liver [51]. However, caution has to be taken with buprenorphine as it can suppress respiration, cause sleepiness or slow down the recovery of anaesthesia [50].

NSAID’s such as carprofen (Rimadyl, Pfizer), ketoprofen (Rofenid, Sanofi-Aventis), ketorolac (Taradyl, Roche), and meloxicam (Mobic, Boehringer Ingelheim) are also useful in laboratory animals, all the more since they exhibit some pleiotropic effects, such as reduction of fever and inflammation. However, as NSAID’s also have renal side-effects, they are counter-indicated for being used as analgesic during induction of IRI [46].

Shortly after induction of anaesthesia, buprenorphine, diluted in sterile saline, is provided via intraperitoneal injection. In general, it is not necessary to provide additional analgesia during the post-operative care since mouse behaviour does not show significant signs of distress after the initial dose of buprenorphine [43].

#### Ischemia time

Renal ischemia time is an important determinant of AKI severity and subsequent renal pathology [52,53]. The most commonly used ischemia times for BIRI and UIRI with contralateral nephrectomy are 30 minutes in mice and 45 minutes in rats. For UIRI without nephrectomy 30, 45 and 60 minutes of ischemia are most frequently used in mice and 45 minutes in rats.

Ischemia induces inhibition of active ion transmembrane transport because of depletion of intracellular energy stores, resulting in increased ion and water influx, causing cell swelling and oedema. The influx of water and ions results in local hemoconcentration because of the transmigration of water into the cells, which causes increased blood viscosity. Stiffening of leukocytes and the increased leukocyte-endothelial cell and neutrophil-neutrophil interactions further impairs blood flow properties, which hinder the restoration of microvascular blood flow upon reperfusion, also known as the “no reflow” phenomenon. Severity of the no reflow phenomenon and the cellular oedema are dependent on the time of ischemia [53–55]. The duration of ischemia necessary to induce a progressive and persistent renal injury depends on the properties of the vascular clip, mouse strain [56], gender [57], mouse weight (as fat tissue can insulate) and thus needs to be optimized and standardized empirically.

#### Body temperature during ischemia-reperfusion

Another important determinant of renal outcome after renal ischemia-reperfusion is body temperature during ischemia. The effect of body temperature on the severity of acute IRI is connected to the body metabolism, and relates to three different processes: 1) higher body temperature during ischemia results in a more severe decrease of intracellular energy stores, 2) the concentration of degradation products inosine and hypoxanthine increases with increasing body temperature during ischemia, which results in increased production of free radicals upon reperfusion, and 3) increasing the body temperature during ischemia produces an increased damage of cell membranes [13,52,58]. It is known that hypothermia during experimental IRI provides renal protection, as it delays degradation processes and extends cell tolerance to ischemic stress [58]. In addition, hypothermia reduces inflammatory processes and limits the increase in vascular permeability [59]. Thus, temperature control during ischemia is one of the most important aspects of IRI models, and is necessary for reproducibility, yet far more difficult to standardize than ischemia time. Appropriate temperature control equipment is necessary since lack of active temperature control exposes the animals to daily and seasonal variations in room temperature and air drafts, even when placed on a heating mat [43]. The latter is nicely illustrated by experimental work of Delbridge et al. demonstrating the difference in AKI severity, as measured by serum creatinine, when rats underwent BIRI on either a heating mat without temperature control, heating mat with rectal temperature-control or without heating mat [13]. These and our own observations illustrate that monitoring body temperature during the procedure is of particular importance, as the body temperature of the animal needs to remain stable during ischemia, preferably up to the moment the animal regains consciousness. With regard to adequate temperature control, it is important to keep in mind that the body temperature of animals, sedated at room temperature can decrease several degrees following administration of ketamine and xylazine [44]. Also, heating the animal with a heat lamp and heating mat needs to be closely monitored as body temperature can spike at 38°C or higher, increasing the variability within the group. As an alternative, a neonatal incubator provides a more stable temperature controlled environment which avoids body temperature spikes and prevents the anaesthesia-associated temperature drop when animals are put inside immediately after sedation (own observations). Overall, it should be noted that temperature settings depend on the lab environment, the mouse strain, and mouse weight (as fat tissue can insulate) and therefore require an empiric optimization and standardization.

### Experimental evaluation of unilateral ischemia-reperfusion in mice

#### Effect of body temperature during ischemia on fibrotic outcome

**Fig 1:**
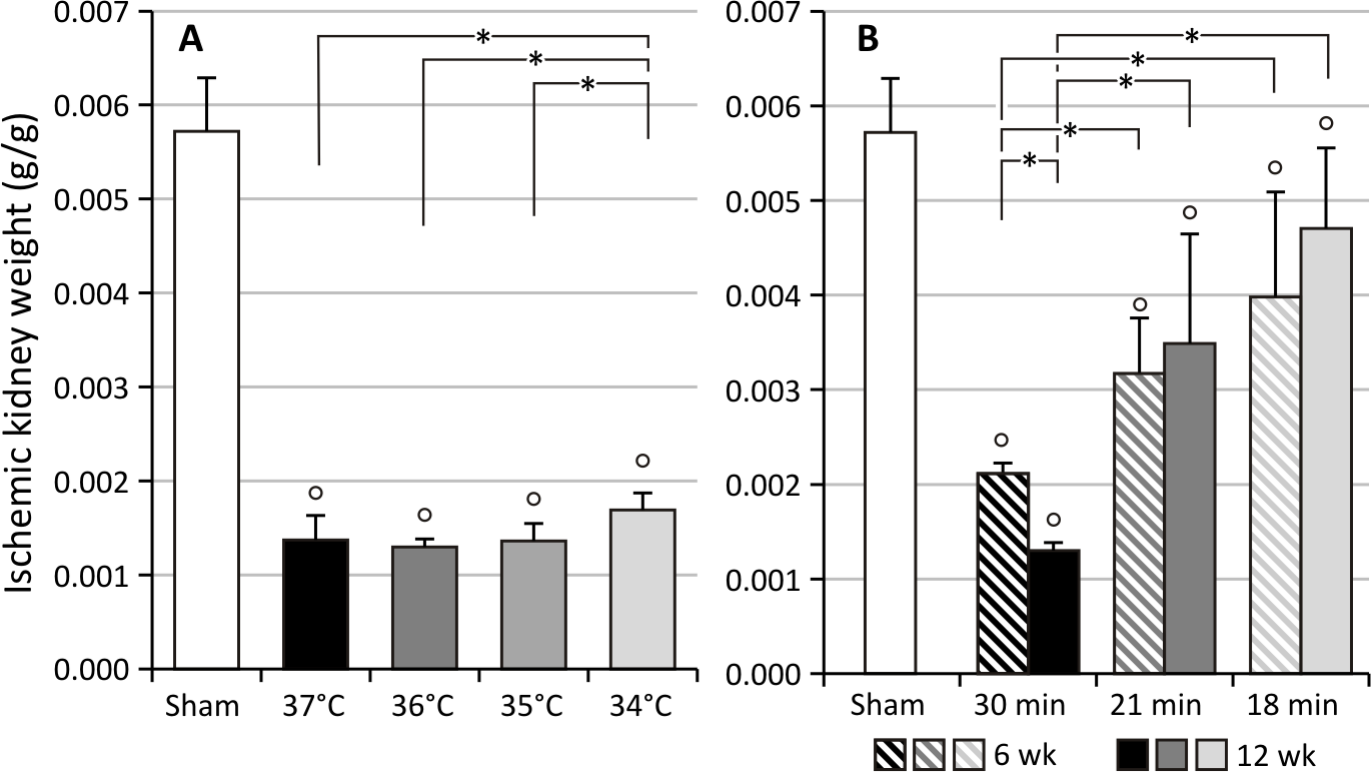
Ischemic kidney weight at euthanasia. Kidney weights are corrected for body weight. *: p<0.05, °: p<0.05 vs. Sham. **A:** UIRI was performed for 30 minutes at 37°C (n = 5), 36°C (n = 4), 35°C (n = 10) or 34°C (n = 5) and animals were euthanized 12 weeks after UIRI. UIRI results in a significant reduction of renal mass (p<0.05) at all temperature conditions tested. **B:** UIRI was performed for 30, 21 or 18 minutes at 36°C and animals were euthanized 6 weeks (resp. n = 5, n = 12, n = 6) and 12 weeks (resp. n = 4, n = 5, n = 10) after UIRI. UIRI causes an ischemia time-dependent reduction in renal mass, with a significantly more severe reduction in renal mass with longer ischemia times. The bars are the means ± s.d. The data were analysed using a two-tailed Mann-Whitney U test.

**Fig 2:**
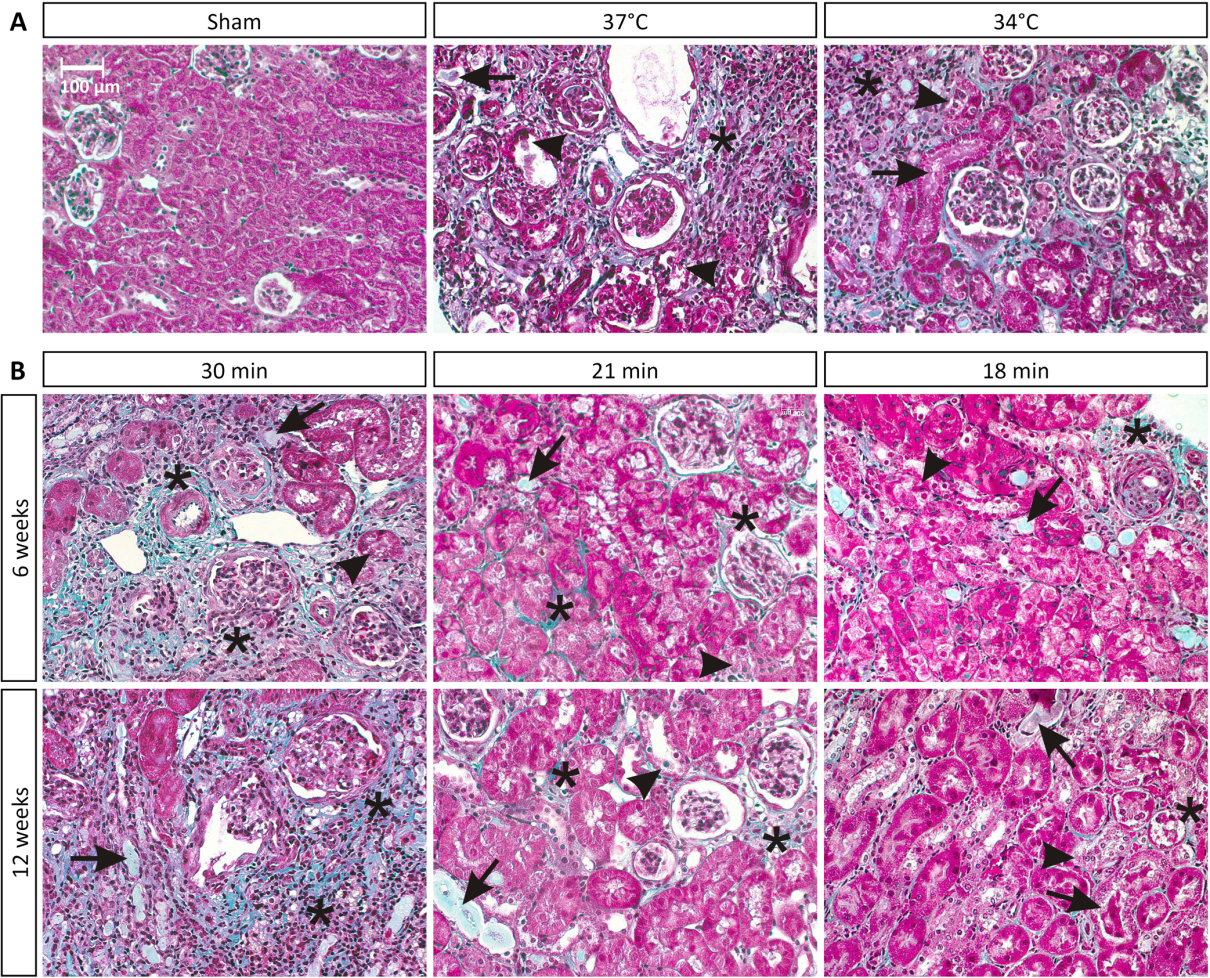
Photos of Masson’s stained slides of ischemic kidney tissue. The images shown are representative of the group. Masson’s stain showed prominent renal damage and severe loss of structure, with necrotic cells (arrowhead), casts or intraluminal debris (arrow), inflammatory infiltration and fibrosis (*). Blue stain represents extracellular matrix deposition (i.e. fibrosis). Magnification: 200x. **A:** Effect of body temperature on long-term fibrotic outcome 12 weeks after UIRI. **B:** Effect of ischemia time on long-term fibrotic outcome 6 and 12 weeks after UIRI.

**Fig 3:**
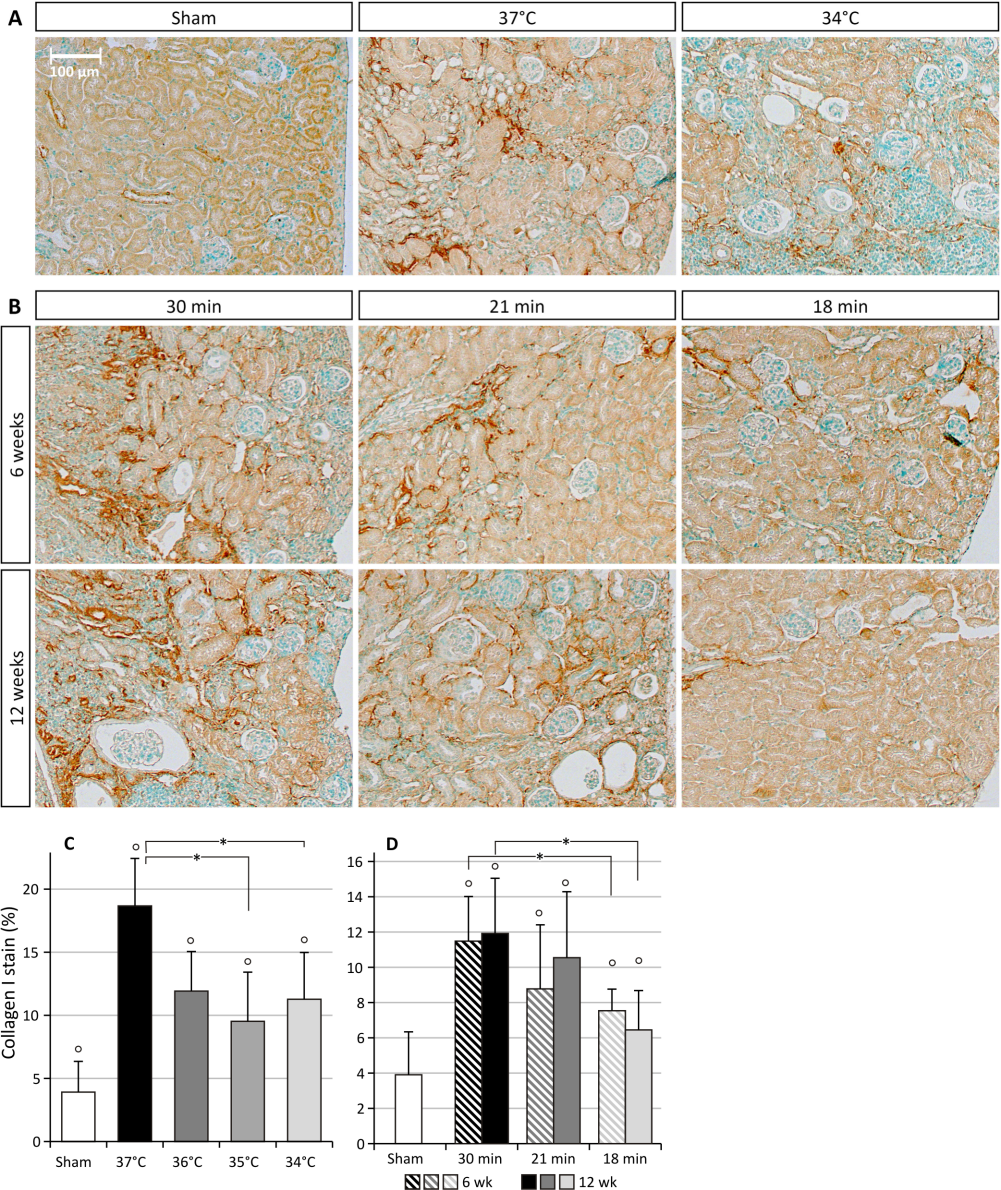
Collagen I immunostaining in the ischemic kidneys. *: p<0.05, °: p<0.05 vs. Sham. **A:** Effect of body temperature on long-term collagen I deposition in the ischemic kidney, 12 weeks after UIRI (magnification: 100x). **B:** Effect of ischemia time on long-term collagen I deposition in the ischemic kidney, 6 and 12 weeks after UIRI (magnification: 100x). **C:** UIRI was performed for 30 minutes at 37°C (n = 5), 36°C (n = 4), 35°C (n = 10) or 34°C (n = 5) and animals were euthanized 12 weeks after UIRI. Collagen I deposition seems to be dependent on body temperature during ischemia: more collagen I deposition after UIRI at higher body temperatures. **D:** UIRI was performed for 30, 21 or 18 minutes at 36°C and animals were euthanized 6 weeks (resp. n = 5, n = 12, n = 6) and 12 weeks (resp. n = 4, n = 5, n = 10) after UIRI. Collagen I deposition seems to be ischemia time-dependent: more collagen I deposition after longer ischemia times. The bars are the means ± s.d. The data were analysed using a two-tailed Mann-Whitney U test.

**Fig 4:**
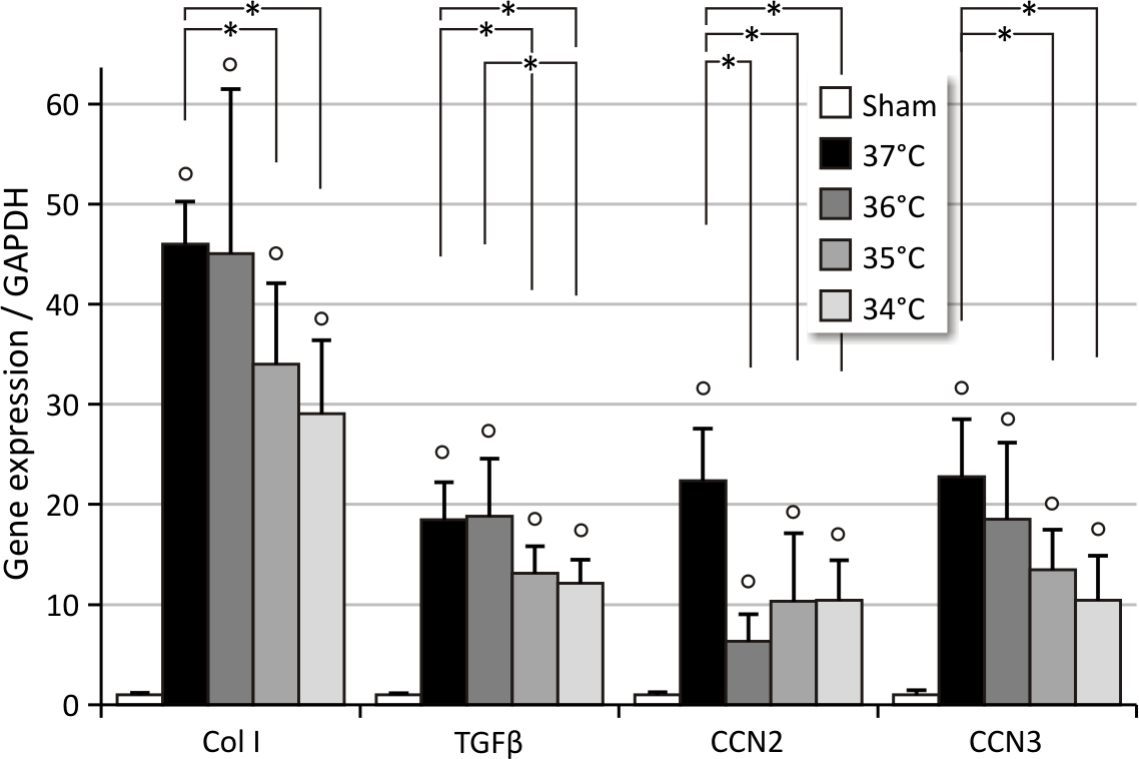
Relative quantification of long-term IRI-induced expression of fibrosis-related genes. Core body temperature during ischemia determines degree of long-term fibrotic outcome. *: p<0.05, °: p<0.05 vs. Sham. UIRI was performed for 30 minutes at 37°C (n = 5), 36°C (n = 4), 35°C (n = 10) or 34°C (n = 5) and animals were euthanized 12 weeks after UIRI. Twelve weeks after UIRI, a significant increase in gene expression of fibrosis-related genes *Col I*, *TGFβ*, *CCN2* and *CCN3* was observed in renal cortex tissue in all core body temperature conditions tested. The expression of these genes is also temperature-dependent: higher expression with higher temperature during ischemia. The bars are the means ± s.d. The data were analysed using a two-tailed Mann-Whitney U test.

Unilateral renal ischemia-reperfusion injury (UIRI) results in a significant reduction of renal mass (p<0.05) at all temperature conditions tested. As depicted in Fig 1A, UIRI at 37°C caused a ±75% reduction (p<0.05) in renal mass, whereas the mildest temperature condition tested, i.e. 34°C, also caused a less pronounced (p<0.05) but still severe reduction in renal mass (±70%). Masson’s stain showed prominent renal damage and severe loss of structure, atrophic renal cortex with disruption of tubular architecture, marked tubule necrosis and intratubular casts, and extensive interstitial inflammatory infiltration (Fig 2A). Quantification of fibrosis by collagen I immunostaining demonstrated an increased deposition of collagen I for all body temperatures under study as compared to sham (p<0.05), with a more pronounced increase in collagen I staining for UIRI at 37°C as compared to the lower body temperatures (35°C and 34°C) (p<0.05) (Fig 3C). As shown in Fig 4, 12 weeks after UIRI a significant increase in gene expression of fibrosis-related genes *Col I*, *TGFβ*, *CCN2* and *CCN3* was observed in renal cortex tissue in all core body temperature conditions tested as compared to sham (p<0.05). The long-term UIRI-induced expression of these genes is also temperature-dependent: higher expression with higher temperature during ischemia (37°C and 36°C vs. 35°C and 34°C; p<0.05) (Fig 4).

#### Effect of body temperature during ischemia on long-term expression of inflammatory and tubular injury markers

**Fig 5:**
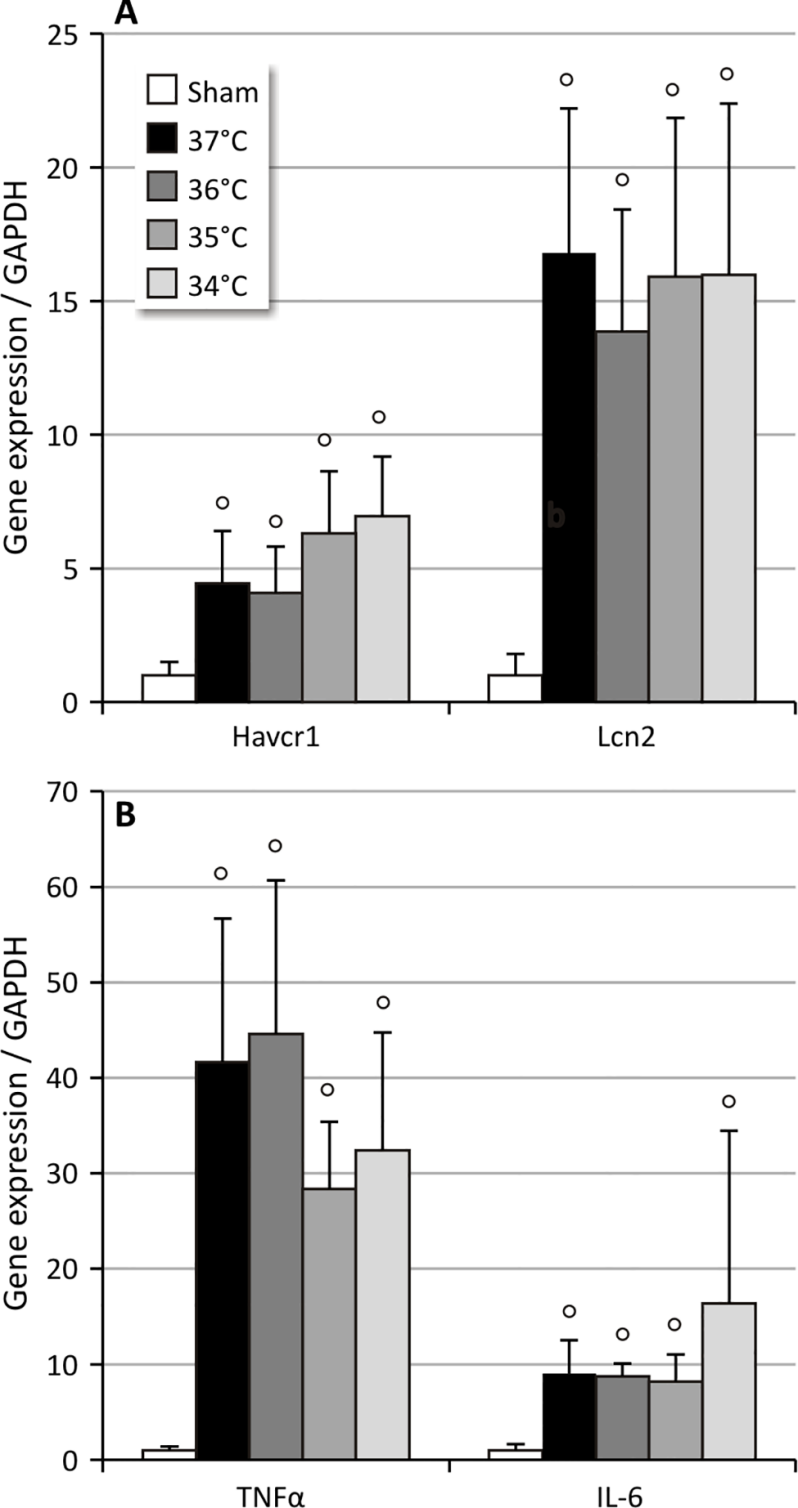
Relative quantification of long-term IRI-induced expression of tubular injury and inflammatory markers. °: p<0.05 vs. Sham UIRI was performed for 30 minutes at 37°C (n = 5), 36°C (n = 4), 35°C (n = 10) or 34°C (n = 5) and animals were euthanized 12 weeks after UIRI. **A:** Twelve weeks after UIRI, a significant increase in gene expression of tubular injury markers *Havcr1* (KIM-1) and *Lcn2* (NGAL) was observed in renal cortex tissue in all core body temperature conditions tested. B: Twelve weeks after UIRI, a significant increase in gene expression of inflammatory cytokines *TNFα* and *IL-6* was observed in renal cortex tissue in all core body temperature conditions tested. The bars are the means ± s.d. The data were analysed using a two-tailed Mann-Whitney U test.

Analysis of gene expression of hepatitis A virus receptor 1 *(Havcr1*; T cell immunoglobulin mucin protein 1 (TIM-1)-producing gene in mice; KIM-1, human homolog) and lipocalin 2 *(Lcn2*; neutrophil gelatinase-associated lipocalin; NGAL) as markers for sustained tubular injury showed a significant upregulation of both markers (p<0.05) at all temperature conditions tested (Fig 5A). In addition, expression of the inflammatory cytokines tumor necrosis factor (TNF)-α and interleukin (IL)-6 were significantly higher (p<0.05) at all temperature conditions tested (Fig 5B). However, no temperature-dependence was observed for the tubular injury markers and inflammatory cytokines gene expression.

#### Effect of ischemia time on fibrotic outcome

**Fig 6:**
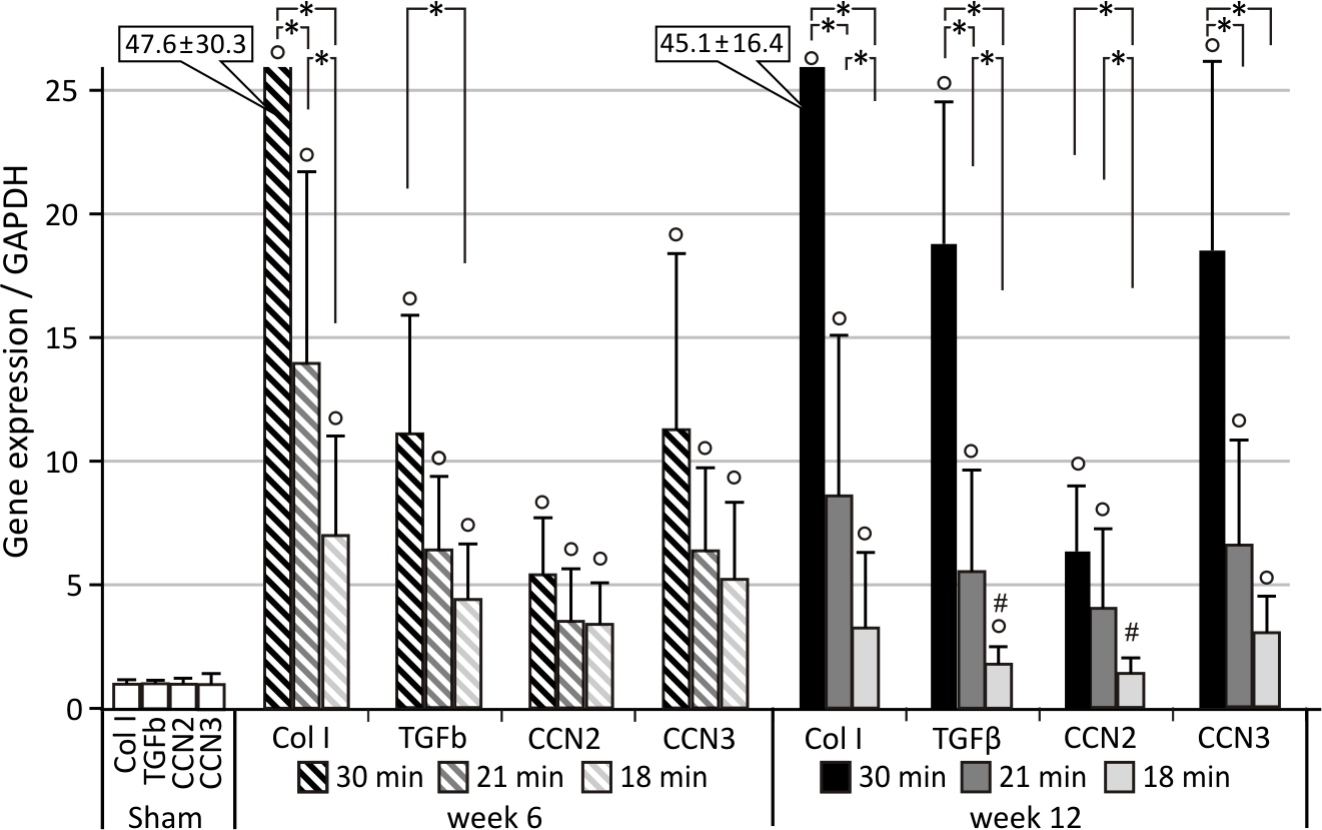
Relative quantification of long-term IRI-induced expression of fibrosis-related genes. Duration of ischemia determines degree of long-term fibrotic outcome. *: p<0.05, °: p<0.05 vs. Sham, #: p<0.05 vs. week 6. UIRI was performed for 30, 21 or 18 minutes at 36°C and animals were euthanized 6 weeks (resp. n = 5, n = 12, n = 6) and 12 weeks (resp. n = 4, n = 5, n = 10) after UIRI. Six weeks after 30, 21 and 18 minutes of UIRI, a significant increase in gene expression of fibrosis-related genes *Col I*, *TGFβ*, *CCN2* and *CCN3* was observed. 12 weeks after 30 and 21 minutes of UIRI, although not statistically significant, a further increase in gene expression of these genes is observed. However, 12 weeks after 18 minutes of UIRI, a trend to decreased gene expression of *Col I* and *CCN3* and a significant decrease in of *TGFβ* and *CCN2* is observed. The bars are the means ± s.d. The data were analysed using a two-tailed Mann-Whitney U test.

As depicted in Fig 1B, UIRI caused a significant reduction in renal mass at all ischemia time-conditions tested as compared to sham. In addition, longer ischemia times induce a more severe reduction in renal mass: 30 minutes UIRI caused a ±75% reduction in renal mass (p<0.05), whereas the mildest ischemia time condition tested, i.e. 18 minutes UIRI, caused a ±20% reduction in renal mass (p<0.05). The severity of histologic renal damage is dependent on ischemia time: 30 minutes of UIRI caused prominent renal damage and severe loss of structure (Fig 2B), as was also seen in the previous experiment on the effect of body temperature during ischemia. On the other hand, 6 and 12 weeks after 18 minutes of UIRI, renal tissue had a more or less normal appearance with some intratubular casts and necrotic tubuli (Fig 2B). Quantification of fibrosis by collagen I immunostaining shows an ischemia time-dependent effect, with significantly less collagen I staining after 18 minutes UIRI as compared to 30 minutes, both at week 6 and 12 (p<0.05; Fig 3D). In addition, a tendency towards progression of renal fibrosis from week 6 to week 12 is seen with 30 and 21 minutes of UIRI. However, the mildest ischemia time-condition, i.e. 18 minutes UIRI, shows tendency towards reduction in collagen I deposition from week 6 to week 12 (Fig 3D). As shown in Fig 6, 12 weeks after 30, 21 and 18 minutes UIRI, a significant increase in gene expression of fibrosis-related genes *Col I*, *TGFβ*, *CCN2* and *CCN3* was observed as compared to sham (p<0.05). At week 12, the increase in gene expression of these fibrosis-related genes is less pronounced with shorter ischemia times, i.e. 21 and 18 minutes UIRI, as compared to 30 minutes UIRI (p<0.05). Also, 12 weeks after 18 minutes UIRI, expression of the pro-fibrotic genes *Col I*, *TGFβ* and *CCN2* is even lower as compared to 21 minutes UIRI (p<0.05). There is a tendency towards higher gene expression of the fibrosis-related genes 12 weeks after 30 and 21 minutes UIRI as compared to 6 weeks (p>0.05). However, 12 weeks after 18 minutes of UIRI, gene expression of *TGFβ* and *CCN2* is significantly lower as compared to week 6 (p<0.05).

#### Effect of ischemia time on long-term expression of inflammatory and tubular injury markers

**Fig 7:**
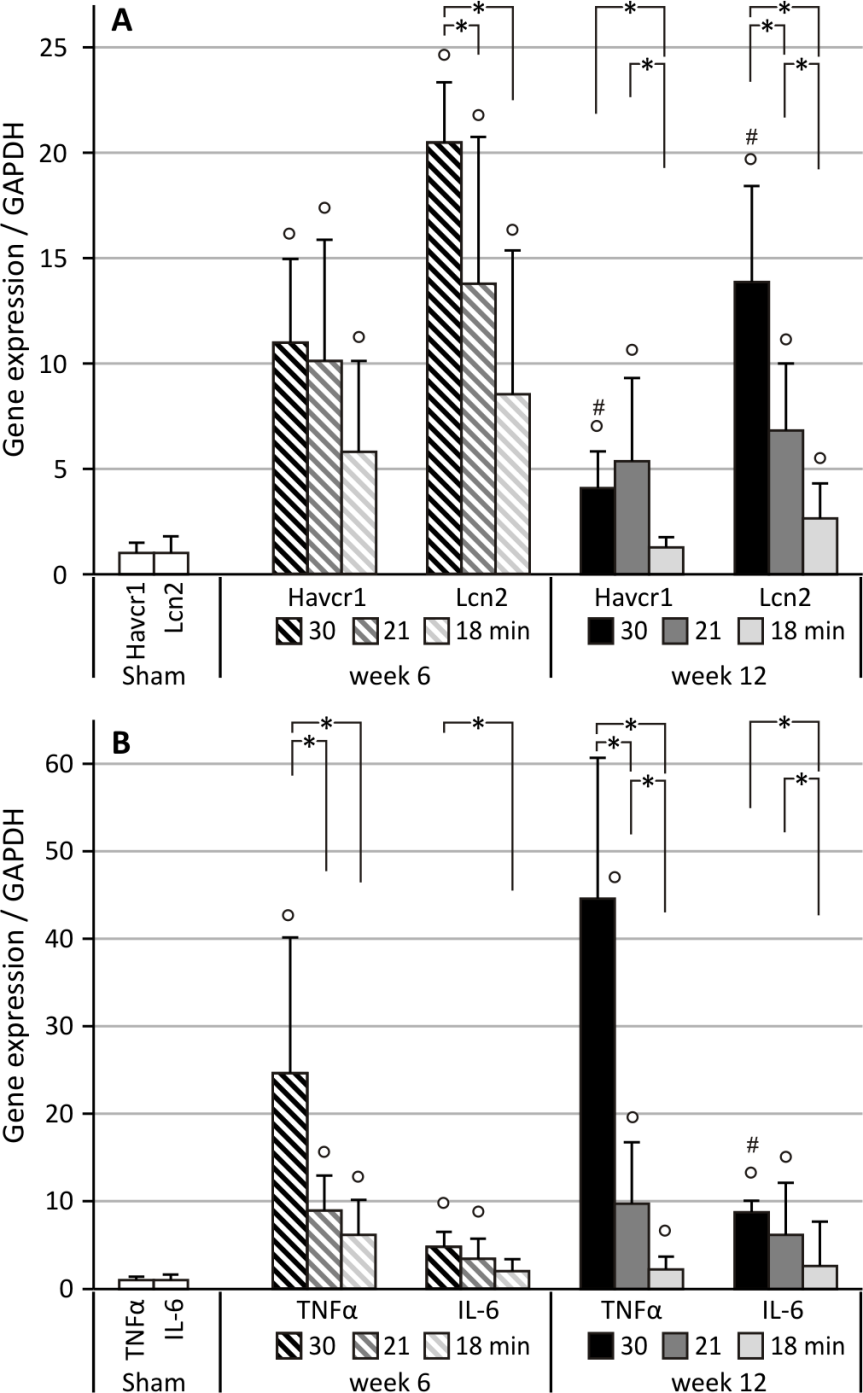
Relative quantification of long-term IRI-induced expression of tubular injury and inflammatory markers. *: p<0.05, °: p<0.05 vs. Sham, #: p<0.05 vs. week 6. UIRI was performed for 30, 21 or 18 minutes at 36°C and animals were euthanized 6 weeks (resp. n = 5, n = 12, n = 6) and 12 weeks (resp. n = 4, n = 5, n = 10) after UIRI. **A:** Six weeks after 30, 21 and 18 minutes of UIRI, a significant increase in gene expression of tubular injury markers *Havcr1* (KIM-1) and *Lcn2* (NGAL) was observed. At 12 weeks after UIRI, upregulation of these markers is ischemia-time dependent, with higher upregulation with longer ischemia times. **B:** Six weeks after 30, 21 and 18 minutes of UIRI, a significant increase in gene expression of inflammatory cytokines *TNFα* and *IL-6* was observed. In addition, short ischemia times, i.e. 18 minutes of UIRI, induced significantly lower gene expression of these markers. At 12 weeks after UIRI, upregulation of these inflammatory cytokines shows an ischemia-time dependent effect, with shorter ischemia times inducing less upregulation of gene expression of these inflammatory markers.

As shown in Fig 7A, 6 weeks after 30, 21 and 18 minutes UIRI, a significant increase in gene expression of the tubular injury marker *Havcr1* (KIM-1) was observed as compared to sham (p<0.05). At week 12, expression of *Havcr1* is reduced after 30 minutes of UIRI as compared to week 6. Also, the mildest ischemia-time condition (18 minutes of UIRI) induced a significant lower upregulation of *Havcr1* expression as compared to the most severe condition (30 minutes of UIRI) at week 12 (p<0.05). Upregulation of the gene expression of the tubular injury marker *Lcn2* (NGAL) shows an ischemia time-dependent effect, with significantly reduced upregulation after 18 and 21 minutes of UIRI as compared to 30 minutes at week 6 (p<0.05; Fig 7A). Also, as for *Havcr1*, the expression of *Lcn2* is reduced 12 weeks after 30 minutes of UIRI as compared to week 6 (p<0.05). In addition, at week 12, expression of *Lcn2* is significant lower after 21 minutes of UIRI as compared to 30 minutes. Likewise for 18 minutes of UIRI as compared to 21 and 30 minutes. As shown in Fig 7B, 6 weeks after 30, 21 and 18 minutes of UIRI, a significant increase in gene expression of the inflammatory cytokines *TNFα* and *IL-6* was observed as compared to sham (p<0.05). Shorter ischemia times, i.e. 21 and 18 minutes, induced significant lower upregulation of *TNFα* and *IL-6* (p<0.05) (Fig 7B). At week 12, upregulation of *TNFα* shows an ischemia-time dependent effect, with significantly reduced upregulation after 21 minutes of UIRI as compared to 30 minutes, and likewise for 18 minutes of UIRI as compared to 21 and 30 minutes. Gene expression of *IL-6* is significant higher at week 12 as compared to week 6 after 30 minutes of UIRI. In addition, 12 weeks after 18 minutes of UIRI, i.e. the mildest condition, gene expression of *IL-6* is significantly lower as compared to both 30 and 21 minutes of UIRI (Fig 7B).

## Discussion

Amongst the realm of models to study or intervene with the development of CKD, IRI is a rarely used model [25,60,61]. Yet, together with nephrotoxic injury from drugs (poly pharmacy, radiocontrast drugs, poison, or metals), ischemia (hypoperfusion after surgery, bleeding, dehydration, shock, or sepsis) is a major aetiology in human AKI [62,63]. In addition, recent clinical studies clearly demonstrate a pathological link between AKI and CKD. The hazard ratio for developing ESRD in patients with AKI without previous CKD is 13.0 [64]. Delayed graft function following renal transplantation, dialysis-requiring acute renal failure, old age and incomplete recovery from AKI are associated with an increased risk for renal nephropathy and progression to CKD [65–68]. Experimental work on mechanisms underlying progression from AKI to CKD in ischemic and renal ablation models indicates that a persistent inflammatory response [27,67], alterations in renal microvasculature [31,69,70] and derangements of the endocrine response and abnormalities in circulating mediators [2] may contribute to progressive injury and lack of recovery. Hence, IRI is a clinically relevant model to study the AKI to CKD connection. Most of the experimental research on CKD and fibrosis is performed in the unilateral ureter obstruction model. Although undoubtedly valuable, this model is a correlate for a rather rare cause of human renal disease [60–62]. Here, we describe an ischemic mouse model of acute to chronic kidney injury with minimal mortality and very consistent development of fibrosis in the injured kidney, i.e. unilateral ischemia-reperfusion without contralateral nephrectomy. In this model, we evaluated the impact of the two main determinants of acute ischemic injury, i.e. core body temperature during and duration of ischemia, on long-term fibrotic outcome and concomitant expression of tubular injury and inflammatory markers. In addition to demonstrating that this model is very straightforward for inducing progressive renal histological decay, we also provide detailed practical considerations on the technical procedure of ischemia-reperfusion.

The primary aim of this manuscript was to provide evidence that UIRI is a suitable animal model to study the progression from acute to chronic kidney injury. A macroscopic parameter indicative of progressive fibrotic renal lesions is a reduction in renal mass [29,71]. In accordance with this, our data show a significant reduction in renal mass at all conditions tested (Fig 1). Histological analysis, by means of Masson’s trichrome stain and collagen I immunostaining, reflect the expected clinically relevant histopathology of CKD [29,72,73], characterized by the presence of tubular casts and debris, atrophic tubuli, ongoing inflammation, and tubulo-interstitial fibrosis (Figs 2 and 3). Complementary to the histological analysis, the expression of a panel of fibrosis-related genes was determined by qPCR, i.e. *col I*, an extracellular matrix component; *TGFβ*, an important pro-inflammatory and cell proliferative cytokine; and *CCN2* and *CCN3*, growth factors. In all currently investigated conditions of ischemia, a significant increase in gene expression was observed for all fibrosis genes under study (Figs 4 and 6).

Since our model is initiated by an acute ischemic injury, we also investigated whether the expression of early tubular injury markers KIM-1 and NGAL was still elevated on the long-term. Indeed, we confirmed that renal expression of these markers remained increased in ischemia-induced progressive renal disease (Figs 5A and 7A), as was previously reported in UUO and CDDP-induced fibrosis [74–76]. In addition, we observed an ischemia-time dependent effect, in particular for NGAL and less pronounced for KIM-1, such that long-term expression of the tubular injury markers increased with longer ischemia times (Fig 7A). These findings are in accordance to the findings of van Timmeren et al. who found an association between tubular KIM-1 expression and interstitial fibrosis in renal biopsies from patients with a variety of renal pathologies [77]. In our experiment, although upregulation of NGAL and KIM-1 persisted up to week 12, expression at week 6 was higher (Fig 7A). Interestingly, a similar decreased expression from week 6 to week 12 after UIRI without contralateral nephrectomy has been reported previously [78]. The fact that it has been demonstrated in renal biopsies that completely atrophic (as well as normal) tubules do not express KIM-1, might explain the decreased expression at week 12 as compared to week 6 (Fig 7A) [77,79].

Since it is known that the model of ischemia-reperfusion features a pronounced inflammatory response, we examined the gene expression of 2 inflammatory cytokines, i.e. TNFα and IL-6, that have already been shown to be upregulated both after an acute injury and in the chronic renal injury phase [29,80,81]. We confirmed that renal expression of these inflammatory cytokines remained increased in ischemia-induced progressive renal disease (Figs 5B and 7B). As in the case of the tubular injury markers, we observed an ischemia-time dependent effect, in particular for TNFα and less pronounced for IL-6, with higher expression of the inflammatory cytokines with longer ischemia times (Fig 7B). Thus, our results show that an acute unilateral ischemic insult results in long-term, active and progressive fibrotic lesions, rendering UIRI without contralateral nephrectomy a suitable model to study the histopathological progression from acute to chronic kidney injury.

Next, we set out to investigate to what extent alterations in body temperature during ischemia and ischemia time influence long-term fibrotic outcome in the UIRI model. Hereto we performed UIRI in a range of conditions commonly used in short-term UIRI experiments, i.e. variations in body temperature from 34°C-37°C and variations in ischemia time from 18–30 minutes. In transplant biology, it is known that both warm ischemia time, i.e. duration between clamping of blood flow and prelevation of the kidney, and cold ischemia time, i.e. duration of extra-corporal ischemia time, are risk factors for delayed graft function and adverse outcomes [34,82,83]. The results of the experiments described in this paper demonstrate that both determinants influence the severity and natural course of the subsequent renal pathology that develops after ischemic AKI. However, taking into account the expression of the tubular injury markers, inflammatory cytokines and fibrosis-related genes, body temperature during ischemia should be particularly thought of as an important factor of variance within the model, and should not be taken lightly in view of standardization of the ischemia-reperfusion model. Ischemia time, on the other hand, is the main factor that determines the severity of the long-term fibrotic outcome. This is a finding that is also true for other variants of the IRI model (bilateral IRI and unilateral IRI with contralateral nephrectomy) [13,43,52,53,58]. Contrary to these other variants of the IRI model, where spontaneous recovery of the ischemic kidneys is seen despite similar ischemia-conditions [25,84], it should be noted that all ischemia conditions tested in our study, both severe and mild, induced renal fibrosis consistently. Only 18 minutes of ischemia, which generally is a rather mild ischemia condition, did not appear to result in progressive fibrosis.

Nevertheless, as higher core body temperature during ischemia and/or longer ischemia times both cause a more severe reduction in renal mass (Fig 1), the model of UIRI can be considered a tuneable model for either acute to chronic kidney injury or reversibility of the acute injury. Indeed, we also showed that depending on the severity of the ischemic insult, i.e. high (37°C) vs. lower body temperature (34°C) and 30 minutes vs. 18 minutes of UIRI, either progression or reversal of the renal pathology can be achieved. The latter is in accordance with findings from others who also showed that short ischemia times (<18 minutes of warm ischemia) induced reversible renal injury without long-term effects [85,86].

In view of the above, it is not surprising that the increase in expression of fibrosis-related genes also depends on the duration of ischemia and core body temperature during ischemia, with higher body temperatures (37°C and 36°C) having a more pronounced effect than lower temperatures (35°C and 34°C) as reflected by the higher increase in gene expression of *Col I*, *TGFβ*, *CCN2*, *CCN3* at higher body temperature during ischemia and longer ischemia times (Figs 4 and 6). Besides body temperature (to a certain extent) and duration of ischemia as determinants of renal pathology, a number of factors must also be taken into account as possible sources of variation, such as strain [56], gender [57], age, anaesthesia [49] and pre-operative preparation of the animal. However, in a consistent experimental setup, these factors of variation are expected to be standardized such that fine-tuning of the ischemia conditions only relies on duration of ischemia and body temperature.

Given the fact that a variety of factors greatly influence the outcome after IRI, our secondary aim was to provide a technical scaffold of the complete procedure and provide some guidelines that are of particular interest for starters as well as create awareness for established researchers when comparing and interpreting their data with historical data from others. Before initiating research with the renal ischemia-reperfusion model in whatever form, be it unilateral or bilateral, pilot experiments need to be performed to optimize surgical procedures and standardize ischemia conditions (i.e. duration and body temperature). Although our experiments allow to put forward conditions for the induction of various degrees of renal injury, these are only directive and may substantially differ from conditions applied by others using other strains or slightly different procedures [43,87]. Therefore, before applying conditions and procedures reported by others, it is recommended to, at first instance, reproduce their validated findings and observations. Depending on the research focus, such observations can either be biochemical (creatinine, BUN, proteinuria), molecular (fibrosis-related gene or protein expression) or histological (e.g. percent positivity on collagen I immunostaining or the number of Ki67 positive nuclei per 400x field). If results (including mortality rate) diverge substantially (>20%) from the selected reference data, additional fine-tuning is warranted. Since our data indicate that ischemia time, rather than core body temperature during ischemia, influences the extent of the fibrotic outcome (Figs 1–3), fine-tuning is preferentially done by adjusting the duration of ischemia, e.g. in 5 minute steps at first. It needs to be stressed however, that this does not rule out the importance of tight temperature control during the ischemia-reperfusion procedure, as this is still an important source of variation. For accurate results we recommend to use a heating pad with feedback system through a rectal temperature probe along with a heat lamp. To further increase temperature control, we found additional benefit in keeping the mice in a neonate incubator during ischemia.

In view of the above, it should be mentioned that applying reported ischemia-reperfusion conditions or procedures remains a challenge. This is mainly due to the overall lack of consensus on which information regarding the conditions of the IRI-model, e.g. body temperature during, ischemia time, method of temperature control, strain, gender and age of the animals, etc., should minimally be reported. This complicates the interpretation of results from different laboratories and often does not allow reproduction or unbiased comparison of data. To this end we provide some recommendations to obviate this important issue. *First*, it is important to mention whether ischemia-reperfusion is conducted bilaterally or unilaterally, and in case of unilateral IRI, whether or not a contralateral nephrectomy is performed, and, if so, at which time following ischemia. *Second*, when referring to previous reported methods, authors have to ensure that these references cover the required information that allows a good insight in the details of the model. *Third*, authors often do not report the temperature at which ischemia-reperfusion is conducted, or only mention the temperature of the operating mat, which differs from the body temperature of the animal. *Fourth*, the strain and number of animals included per group, as well as mortality rate should be mentioned, as this is an additional indicator of severity of the insult. *Last*, experimental end-points (e.g. time-point of euthanasia, criteria for early euthanasia) should be mentioned in the methods-section.

In conclusion, we demonstrate that UIRI without nephrectomy is a very robust model for induction of long-term tubulo-interstitial fibrosis. In addition, we demonstrate that varying the two main determinants of IRI induced AKI, i.e. body temperature during and duration of ischemia, in the unilateral IRI without nephrectomy model allows tuning of these long-term effects. Also, we provide a detailed overview of the technical procedure and highlight some additional factors that influence variation within the UIRI model, such as anaesthesia and pre-and post-operative care. Finally, a number of considerations and recommendations are provided on which crucial information of the IRI-model should be reported to ensure transferability of this technique.

## References

[1] Coresh J, Selvin E, Stevens LA, Manzi J, Kusek JW, Eggers P, et al (2007) Prevalence of chronic kidney disease in the United States. JAMA 298: 2038–2047. 17986697 10.1001/jama.298.17.2038

[2] Chawla LS, Kimmel PL (2012) Acute kidney injury and chronic kidney disease: an integrated clinical syndrome. Kidney Int 82: 516–524. 10.1038/ki.2012.208 22673882

[3] Basile DP, Donohoe D, Roethe K, Osborn JL (2001) Renal ischemic injury results in permanent damage to peritubular capillaries and influences long-term function. Am J Physiol Renal Physiol 281: F887–899. 11592947 10.1152/ajprenal.2001.281.5.F887

[4] Jones J, Holmen J, De Graauw J, Jovanovich A, Thornton S, Chonchol M (2012) Association of complete recovery from acute kidney injury with incident CKD stage 3 and all-cause mortality. Am J Kidney Dis 60: 402–408. 10.1053/j.ajkd.2012.03.014 22541737 PMC3422603

[5] Dhaun N, Webb DJ (2013) The road from AKI to CKD: the role of endothelin. Kidney Int 84: 637–638. 10.1038/ki.2013.158 24080871

[6] Lieberthal W, Nigam SK (2000) Acute renal failure. II. Experimental models of acute renal failure: imperfect but indispensable. Am J Physiol Renal Physiol 278: F1–F12. 10644651 10.1152/ajprenal.2000.278.1.F1

[7] Alpers CE (2014) Animal models of renal disease. Drug Discovery Today: Disease Models 11: 1–4.

[8] Humphreys BD, DiRocco DP (2014) Lineage-tracing methods and the kidney. Kidney Int 86: 481–488. 10.1038/ki.2013.368 24088959 PMC3975825

[9] Adachi T, Sugiyama N, Gondai T, Yagita H, Yokoyama T (2013) Blockade of Death Ligand TRAIL Inhibits Renal Ischemia Reperfusion Injury. Acta Histochem Cytochem 46: 161–170. 10.1267/ahc.13022 24610963 PMC3929614

[10] Basile DP, Martin DR, Hammerman MR (1998) Extracellular matrix-related genes in kidney after ischemic injury: potential role for TGF-beta in repair. Am J Physiol 275: F894–903. 9843906 10.1152/ajprenal.1998.275.6.F894

[11] Kim J, Padanilam BJ (2014) Renal denervation prevents long-term sequelae of ischemic renal injury. Kidney Int. 10.1038/ki.2014.300 PMC4312521 25207878

[12] Gueler F, Gwinner W, Schwarz A, Haller H (2004) Long-term effects of acute ischemia and reperfusion injury. Kidney Int 66: 523–527. 15253702 10.1111/j.1523-1755.2004.761_11.x

[13] Delbridge MS, Shrestha BM, Raftery AT, El Nahas AM, Haylor JL (2007) The effect of body temperature in a rat model of renal ischemia-reperfusion injury. Transplant Proc 39: 2983–2985. 18089305 10.1016/j.transproceed.2007.04.028

[14] Nguan CY, Guan Q, Gleave ME, Du C (2014) Promotion of cell proliferation by clusterin in the renal tissue repair phase after ischemia-reperfusion injury. Am J Physiol Renal Physiol 306: F724–733. 10.1152/ajprenal.00410.2013 24477687

[15] Zager RA, Gmur DJ, Bredl CR, Eng MJ (1989) Degree and time sequence of hypothermic protection against experimental ischemic acute renal failure. Circ Res 65: 1263–1269. 2805243 10.1161/01.res.65.5.1263

[16] Mangino MJ, Murphy MK, Grabau GG, Anderson CB (1991) Protective effects of glycine during hypothermic renal ischemia-reperfusion injury. Am J Physiol 261: F841–848. 1951715 10.1152/ajprenal.1991.261.5.F841

[17] Wang YL, Li G, Zou XF, Chen XB, Liu T, Shen ZY (2013) Effect of autologous adipose-derived stem cells in renal cold ischemia and reperfusion injury. Transplant Proc 45: 3198–3202. 10.1016/j.transproceed.2013.07.061 24182784

[18] del Moral RM, Gomez-Morales M, Hernandez-Cortes P, Aguilar D, Caballero T, Aneiros-Fernandez J, et al (2013) PARP inhibition attenuates histopathological lesion in ischemia/reperfusion renal mouse model after cold prolonged ischemia. ScientificWorldJournal 2013: 486574 10.1155/2013/486574 24319370 PMC3844238

[19] Hosgood SA, Hunter JP, Nicholson ML (2012) Early urinary biomarkers of warm and cold ischemic injury in an experimental kidney model. J Surg Res 174: e85–90. 10.1016/j.jss.2011.10.024 22221604

[20] Hosgood SA, Patel M, Nicholson ML (2013) The conditioning effect of ex vivo normothermic perfusion in an experimental kidney model. J Surg Res 182: 153–160. 10.1016/j.jss.2012.08.001 22940032

[21] Gottmann U, Brinkkoetter PT, Bechtler M, Hoeger S, Karle C, Schaub M, et al (2006) Effect of pre-treatment with catecholamines on cold preservation and ischemia/reperfusion-injury in rats. Kidney Int 70: 321–328. 16760913 10.1038/sj.ki.5001501

[22] Stubenitsky BM, Brasile L, Booster MH, Haisch CE, Kootstra G (2001) Deletrious effect of prolonged cold ischemia on renal function. Transpl Int 14: 256–260. 11512059 10.1007/s001470100326

[23] Ertugrul A, Turkeri LN, Ozyurek M, Ozveri H, Akdas A (1999) Alteration of epidermal growth factor receptor expression following ischaemia of renal tissue. Int Urol Nephrol 31: 611–617. 10755351 10.1023/a:1007100303378

[24] Jani A, Ljubanovic D, Faubel S, Kim J, Mischak R, Edelstein CL (2004) Caspase inhibition prevents the increase in caspase-3, -2, -8 and -9 activity and apoptosis in the cold ischemic mouse kidney. Am J Transplant 4: 1246–1254. 15268725 10.1111/j.1600-6143.2004.00498.x

[25] Skrypnyk NI, Harris RC, de Caestecker MP (2013) Ischemia-reperfusion model of acute kidney injury and post injury fibrosis in mice. J Vis Exp. 10.3791/50495 PMC3854859 23963468

[26] Holderied A, Anders H-J (2014) Animal models of kidney inflammation in translational medicine. Drug Discovery Today: Disease Models 11: 19–27.

[27] Zager RA, Johnson AC, Becker K (2011) Acute unilateral ischemic renal injury induces progressive renal inflammation, lipid accumulation, histone modification, and "end-stage" kidney disease. Am J Physiol Renal Physiol 301: F1334–1345. 10.1152/ajprenal.00431.2011 21921025 PMC3233867

[28] Zager RA, Johnson AC, Andress D, Becker K (2013) Progressive endothelin-1 gene activation initiates chronic/end-stage renal disease following experimental ischemic/reperfusion injury. Kidney Int 84: 703–712. 10.1038/ki.2013.157 23698233 PMC3788861

[29] Ascon M, Ascon DB, Liu M, Cheadle C, Sarkar C, Racusen L, et al (2009) Renal ischemia-reperfusion leads to long term infiltration of activated and effector-memory T lymphocytes. Kidney Int 75: 526–535. 10.1038/ki.2008.602 19092796 PMC2676145

[30] Stokman G, Leemans JC, Claessen N, Weening JJ, Florquin S (2005) Hematopoietic stem cell mobilization therapy accelerates recovery of renal function independent of stem cell contribution. J Am Soc Nephrol 16: 1684–1692. 15829714 10.1681/ASN.2004080678

[31] Horbelt M, Lee SY, Mang HE, Knipe NL, Sado Y, Kribben A, et al (2007) Acute and chronic microvascular alterations in a mouse model of ischemic acute kidney injury. Am J Physiol Renal Physiol 293: F688–695. 17626153 10.1152/ajprenal.00452.2006

[32] Lech M, Avila-Ferrufino A, Allam R, Segerer S, Khandoga A, Krombach F, et al (2009) Resident dendritic cells prevent postischemic acute renal failure by help of single Ig IL-1 receptor-related protein. J Immunol 183: 4109–4118. 10.4049/jimmunol.0900118 19692646

[33] Craddock GN (1976) Species differences in response to renal ischemia. Arch Surg 111: 582–584. 1267609 10.1001/archsurg.1976.01360230082016

[34] Salahudeen AK, Haider N, May W (2004) Cold ischemia and the reduced long-term survival of cadaveric renal allografts. Kidney Int 65: 713–718. 14717946 10.1111/j.1523-1755.2004.00416.x

[35] Zager RA (2009) Uremia induces proximal tubular cytoresistance and heme oxygenase-1 expression in the absence of acute kidney injury. Am J Physiol Renal Physiol 296: F362–368. 10.1152/ajprenal.90645.2008 19036845 PMC2643867

[36] Zager RA, Johnson AC, Lund S (2009) Uremia impacts renal inflammatory cytokine gene expression in the setting of experimental acute kidney injury. Am J Physiol Renal Physiol 297: F961–970. 10.1152/ajprenal.00381.2009 19656911 PMC2775581

[37] Tokuyama H, Hayashi K, Matsuda H, Kubota E, Honda M, Okubo K, et al (2002) Differential regulation of elevated renal angiotensin II in chronic renal ischemia. Hypertension 40: 34–40. 12105135 10.1161/01.hyp.0000022060.13995.ed

[38] Tsuji Y, Ariyoshi A, Sakamoto K (1993) An experimental model for unilateral ischaemic acute renal failure in dog. Int Urol Nephrol 25: 83–88. 8514478 10.1007/BF02552259

[39] Downey P, Tolley DA, Johnston SR, Young M (2001) Ischemia-reperfusion injury after relief of ureteral obstruction: an animal study. J Endourol 15: 209–211. 11325095 10.1089/089277901750134647

[40] Malis CD, Cheung JY, Leaf A, Bonventre JV (1983) Effects of verapamil in models of ischemic acute renal failure in the rat. Am J Physiol 245: F735–742. 6660294 10.1152/ajprenal.1983.245.6.F735

[41] Kassab S, Hamdy H, AbdulGhaffar T, Granger JP (2001) Effects of endothelin-A receptor antagonism on bilateral renal function in renovascular hypertensive rats. Fundam Clin Pharmacol 15: 379–385. 11860525 10.1046/j.1472-8206.2001.xs052.x

[42] Singh AP, Junemann A, Muthuraman A, Jaggi AS, Singh N, Grover K, et al (2012) Animal models of acute renal failure. Pharmacol Rep 64: 31–44. 22580518 10.1016/s1734-1140(12)70728-4

[43] Wei Q, Dong Z (2012) Mouse model of ischemic acute kidney injury: technical notes and tricks. Am J Physiol Renal Physiol 303: F1487–1494. 10.1152/ajprenal.00352.2012 22993069 PMC3532486

[44] Hankenson FC (2013) Critical Care Management for Laboratory Mice and Rats: Taylor & Francis.

[45] Van Zutphen L, Baumans V, Beynen AC (1993) Principles of Laboratory Animal Science: Doses of analgesics for post-operative pain relief.

[46] Kohn DF, Wixson SK, White WJ, Benson GJ (1997) Anesthesia and Analgesia in Laboratory Animals: Elsevier Science.

[47] Gargiulo S, Greco A, Gramanzini M, Esposito S, Affuso A, Brunetti A, et al (2012) Mice anesthesia, analgesia, and care, Part I: anesthetic considerations in preclinical research. ILAR J 53: E55–69. 10.1093/ilar.53.1.55 23382271

[48] Yunos NM, Bellomo R, Hegarty C, Story D, Ho L, Bailey M (2012) Association between a chloride-liberal vs chloride-restrictive intravenous fluid administration strategy and kidney injury in critically ill adults. JAMA 308: 1566–1572. 10.1001/jama.2012.13356 23073953

[49] Lee HT, Ota-Setlik A, Fu Y, Nasr SH, Emala CW (2004) Differential protective effects of volatile anesthetics against renal ischemia-reperfusion injury in vivo. Anesthesiology 101: 1313–1324. 15564938 10.1097/00000542-200412000-00011

[50] Fish R, Danneman PJ, Brown M, Karas A (2011) Anesthesia and Analgesia in Laboratory Animals: Elsevier Science.

[51] Guarnieri M, Brayton C, Detolla L, Forbes-McBean N, Sarabia-Estrada R, Zadnik P (2012) Safety and efficacy of buprenorphine for analgesia in laboratory mice and rats. Lab Anim (NY) 41: 337–343. 23079917 10.1038/laban.152

[52] Kennedy SE, Erlich JH (2008) Murine renal ischaemia-reperfusion injury. Nephrology (Carlton) 13: 390–396. 18844833 10.1111/j.1440-1797.2008.00979.x

[53] Massberg S, Messmer K (1998) The nature of ischemia/reperfusion injury. Transplant Proc 30: 4217–4223. 9865350 10.1016/s0041-1345(98)01397-9

[54] Wilhelm M, Pratschke J, Laskowski I, Tilney N (2003) Ischemia and reperfusion injury. Transplant Rev 17: 140–157.

[55] Kosieradzki M, Rowinski W (2008) Ischemia/reperfusion injury in kidney transplantation: mechanisms and prevention. Transplant Proc 40: 3279–3288. 10.1016/j.transproceed.2008.10.004 19100373

[56] Burne MJ, Haq M, Matsuse H, Mohapatra S, Rabb H (2000) Genetic susceptibility to renal ischemia reperfusion injury revealed in a murine model. Transplantation 69: 1023–1025. 10755573 10.1097/00007890-200003150-00065

[57] Park KM, Kim JI, Ahn Y, Bonventre AJ, Bonventre JV (2004) Testosterone is responsible for enhanced susceptibility of males to ischemic renal injury. J Biol Chem 279: 52282–52292. 15358759 10.1074/jbc.M407629200

[58] Pelkey TJ, Frank RS, Stanley JJ, Frank TS, Zelenock GB, D'Alecy LG (1992) Minimal physiologic temperature variations during renal ischemia alter functional and morphologic outcome. J Vasc Surg 15: 619–625. 1560550

[59] Carattino MD, Cueva F, Zuccollo A, Monti JL, Navarro M, Catanzaro OL (1999) Renal ischemia-induced increase in vascular permeability is limited by hypothermia. Immunopharmacology 43: 241–248. 10596859 10.1016/s0162-3109(99)00095-8

[60] Yang HC, Zuo Y, Fogo AB (2010) Models of chronic kidney disease. Drug Discov Today Dis Models 7: 13–19. 21286234 10.1016/j.ddmod.2010.08.002 PMC3030258

[61] Lim BJ, Yang H-C, Fogo AB (2014) Animal models of regression/progression of kidney disease. Drug Discovery Today: Disease Models 11: 45–51. 25722733 10.1016/j.ddmod.2014.06.003 PMC4337226

[62] Basile DP, Anderson MD, Sutton TA (2012) Pathophysiology of acute kidney injury. Compr Physiol 2: 1303–1353. 10.1002/cphy.c110041 23798302 PMC3919808

[63] Lameire NH, Bagga A, Cruz D, De Maeseneer J, Endre Z, Kellum JA, et al (2013) Acute kidney injury: an increasing global concern. Lancet 382: 170–179. 10.1016/S0140-6736(13)60647-9 23727171

[64] Campbell D, Weir MR (2015) Defining, Treating, and Understanding Chronic Kidney Disease—A Complex Disorder. J Clin Hypertens (Greenwich) 17: 514–527. 25917313 10.1111/jch.12560 PMC8031501

[65] Kimura M, Suzuki T, Hishida A (1999) A rat model of progressive chronic renal failure produced by microembolism. Am J Pathol 155: 1371–1380. 10514419 10.1016/S0002-9440(10)65239-X PMC1867024

[66] Bonventre JV, Yang L (2011) Cellular pathophysiology of ischemic acute kidney injury. J Clin Invest 121: 4210–4221. 10.1172/JCI45161 22045571 PMC3204829

[67] Heung M, Chawla LS (2012) Predicting progression to chronic kidney disease after recovery from acute kidney injury. Curr Opin Nephrol Hypertens 21: 628–634. 10.1097/MNH.0b013e3283588f24 23010757

[68] Spurgeon-Pechman KR, Donohoe DL, Mattson DL, Lund H, James L, Basile DP (2007) Recovery from acute renal failure predisposes hypertension and secondary renal disease in response to elevated sodium. Am J Physiol Renal Physiol 293: F269–278. 17507599 10.1152/ajprenal.00279.2006

[69] Singh P, Ricksten SE, Bragadottir G, Redfors B, Nordquist L (2013) Renal oxygenation and haemodynamics in acute kidney injury and chronic kidney disease. Clin Exp Pharmacol Physiol 40: 138–147. 10.1111/1440-1681.12036 23360244 PMC3710120

[70] Basile DP, Friedrich JL, Spahic J, Knipe N, Mang H, Leonard EC, et al (2011) Impaired endothelial proliferation and mesenchymal transition contribute to vascular rarefaction following acute kidney injury. Am J Physiol Renal Physiol 300: F721–733. 10.1152/ajprenal.00546.2010 21123492 PMC3064142

[71] Kwak W, Jang HS, Belay T, Kim J, Ha YS, Lee SW, et al (2011) Evaluation of kidney repair capacity using 99mTc-DMSA in ischemia/reperfusion injury models. Biochem Biophys Res Commun 406: 7–12. 10.1016/j.bbrc.2011.01.085 21277288

[72] Braun H, Schmidt BM, Raiss M, Baisantry A, Mircea-Constantin D, Wang S, et al (2012) Cellular senescence limits regenerative capacity and allograft survival. J Am Soc Nephrol 23: 1467–1473. 10.1681/ASN.2011100967 22797186 PMC3431409

[73] Feitoza CQ, Goncalves GM, Semedo P, Cenedeze MA, Pinheiro HS, Beraldo FC, et al (2008) Inhibition of COX 1 and 2 prior to renal ischemia/reperfusion injury decreases the development of fibrosis. Mol Med 14: 724–730. 10.2119/2008-00064.Feitoza 18769637 PMC2527343

[74] Kashiwagi E, Tonomura Y, Kondo C, Masuno K, Fujisawa K, Tsuchiya N, et al (2014) Involvement of neutrophil gelatinase-associated lipocalin and osteopontin in renal tubular regeneration and interstitial fibrosis after cisplatin-induced renal failure. Exp Toxicol Pathol 66: 301–311. 10.1016/j.etp.2014.04.007 24912749

[75] Humphreys BD, Xu F, Sabbisetti V, Grgic I, Movahedi Naini S, Wang N, et al (2013) Chronic epithelial kidney injury molecule-1 expression causes murine kidney fibrosis. J Clin Invest 123: 4023–4035. 10.1172/JCI45361 23979159 PMC3755983

[76] Nakagawa S, Nishihara K, Miyata H, Shinke H, Tomita E, Kajiwara M, et al (2015) Molecular Markers of Tubulointerstitial Fibrosis and Tubular Cell Damage in Patients with Chronic Kidney Disease. PLoS One 10: e0136994 10.1371/journal.pone.0136994 26317775 PMC4552842

[77] van Timmeren MM, van den Heuvel MC, Bailly V, Bakker SJL, van Goor H, Stegeman CA (2007) Tubular kidney injury molecule-1 (KIM-1) in human renal disease. The Journal of Pathology 212: 209–217. 17471468 10.1002/path.2175

[78] Ko GJ, Grigoryev DN, Linfert D, Jang HR, Watkins T, Cheadle C, et al (2010) Transcriptional analysis of kidneys during repair from AKI reveals possible roles for NGAL and KIM-1 as biomarkers of AKI-to-CKD transition. Am J Physiol Renal Physiol 298: F1472–1483. 10.1152/ajprenal.00619.2009 20181666

[79] Ichimura T, Bonventre JV, Bailly V, Wei H, Hession CA, Cate RL, et al (1998) Kidney Injury Molecule-1 (KIM-1), a Putative Epithelial Cell Adhesion Molecule Containing a Novel Immunoglobulin Domain, Is Up-regulated in Renal Cells after Injury. Journal of Biological Chemistry 273: 4135–4142. 9461608 10.1074/jbc.273.7.4135

[80] Lech M, Rommele C, Grobmayr R, Eka Susanti H, Kulkarni OP, Wang S, et al (2013) Endogenous and exogenous pentraxin-3 limits postischemic acute and chronic kidney injury. Kidney Int 83: 647–661. 10.1038/ki.2012.463 23325083

[81] Ling H, Chen H, Wei M, Meng X, Yu Y, Xie K (2015) The Effect of Autophagy on Inflammation Cytokines in Renal Ischemia/Reperfusion Injury. Inflammation. 10.1007/s10753-015-0255-5 26412257

[82] Karipineni F, Campos S, Parsikia A, Durinka JB, Chang PN, Khanmoradi K, et al (2014) Elimination of warm ischemia using the Ice Bag Technique does not decrease delayed graft function. Int J Surg 12: 551–556. 10.1016/j.ijsu.2014.04.002 24735894

[83] Haisch C, Green E, Brasile L (1997) Predictors of graft outcome in warm ischemically damaged organs. Transplant Proc 29: 3424–3425. 9414774 10.1016/s0041-1345(97)00963-9

[84] Celie JW, Katta KK, Adepu S, Melenhorst WB, Reijmers RM, Slot EM, et al (2012) Tubular epithelial syndecan-1 maintains renal function in murine ischemia/reperfusion and human transplantation. Kidney Int 81: 651–661. 10.1038/ki.2011.425 22237752

[85] Mishra J, Ma Q, Prada A, Mitsnefes M, Zahedi K, Yang J, et al (2003) Identification of neutrophil gelatinase-associated lipocalin as a novel early urinary biomarker for ischemic renal injury. J Am Soc Nephrol 14: 2534–2543. 14514731 10.1097/01.asn.0000088027.54400.c6

[86] Pang P, Jin X, Proctor BM, Farley M, Roy N, Chin MS, et al (2015) RGS4 inhibits angiotensin II signaling and macrophage localization during renal reperfusion injury independent of vasospasm. Kidney Int 87: 771–783. 10.1038/ki.2014.364 25469849 PMC4382433

[87] Oxburgh L, de Caestecker MP (2012) Ischemia-reperfusion injury of the mouse kidney. Methods Mol Biol 886: 363–379. 10.1007/978-1-61779-851-1_32 22639277

